# Microglia enhances proliferation of neural progenitor cells in an *in vitro* model of hypoxic-ischemic injury

**DOI:** 10.17179/excli2020-2249

**Published:** 2020-07-03

**Authors:** Supanee Chounchay, Stephen C. Noctor, Nuanchan Chutabhakdikul

**Affiliations:** 1 Research Center for Neuroscience, Institute of Molecular Biosciences, Mahidol University, Salaya, Nakhonpathom, 73170, Thailand; 2 Faculty of Physical Therapy, Huachiew Chalermprakiet University, Samut Prakan, 10540, Thailand; 3 Department of Psychiatry and Behavioral Sciences, University of California, Davis, Sacramento, CA, 95817, USA; 4 MIND Institute, University of California, Davis, Sacramento, CA, 95817, USA

**Keywords:** hypoxic-ischemic injury, HIF-1alpha, microglia, neural progenitor cells, proliferation

## Abstract

Microglial cells are the primary immune cells in the central nervous system. In the mature brain, microglia perform functions that include eliminating pathogens and clearing dead/dying cells and cellular debris through phagocytosis. In the immature brain, microglia perform functions that include synapse development and the regulation of cell production through extensive contact with and phagocytosis of neural progenitor cells (NPCs). However, the functional role of microglia in the proliferation and differentiation of NPCs under hypoxic-ischemic (HI) injury is not clear. Here, we tested the hypothesis that microglia enhance NPCs proliferation following HI insult. Primary NPCs cultures were divided into four treatment groups: 1) normoxic NPCs (NN); 2) normoxic NPCs cocultured with microglia (NN+M); 3) hypoxic NPCs (HN); and 4) hypoxic NPCs cocultured with microglia (HN+M). Hypoxic-ischemic injury was induced by pretreatment of the cell cultures with 100 µM deferoxamine mesylate (DFO). NPCs treated with 100 µM DFO (HN groups) for 24 hours had significantly increased expression of hypoxia-inducible factor 1 alpha (HIF-1α), a marker of hypoxic cells. Cell number, protein expression, mitosis, and cell cycle phase were examined, and the data were compared between the four groups. We found that the number of cells expressing the NPCs marker Sox2 increased significantly in the HN+M group and that the number of PH3-positive cells increased in the HN+M group; flow cytometry analysis showed a significant increase in the percentage of cells in the G2/M phase in the HN+M group. In summary, these results support the concept that microglia enhance the survival of NPCs under HI injury by increasing NPCs proliferation, survival, and differentiation. These results further suggest that microglia may induce neuroprotective effects after hypoxic injury that can be explored to develop novel therapeutic strategies for the treatment of HI injury in the immature brain.

## Introduction

Hypoxic-ischemic (HI) injury is a major cause of damage in the immature brain. HI injury is the result of oxygen deprivation due to either reduced blood oxygen levels or diminished blood supply (Vannucci, 1990[48]; Rivkin and Volpe, 1993[34]; Johnston et al., 2001[19]). Hypoxia upregulates hypoxia-inducible factor (HIF), which plays an essential role in cellular adaptation to low blood oxygen levels (Maxwell, 2005[24]; Cavadas et al., 2013[3]). The unique protein HIF-1α is used as a marker of hypoxic cells (Semenza, 2014[38]; Davis et al., 2018[8]). HIF-1α is a heterodimeric transcription factor that functions as the principal regulator of oxygen homeostasis in response to oxygen deprivation (Nizet and Johnson, 2009[28]; Imtiyaz and Simon, 2010[18]; Semenza, 2012[37]). The expression of HIF-1α is regulated by ferric iron (Fe2+) (Greer et al., 2012[15]). Deferoxamine mesylate (DFO) is an iron chelator that is widely used to induce HI injury *in vitro* (Milosevic et al., 2009[25]; Wu and Yotnda, 2011[54]).

HI injury produces neurotoxic events, including neuronal cell death. However, protective or compensatory events can also follow HI, including neurogenesis in the subventricular zone (SVZ) and a stream of double-cortin-positive neuronal cells that migrate into the ischemic brain area (Plane et al., 2004[33]; Ong et al., 2005[30]; Yang and Levison, 2006[57]; Yang et al, 2007[56]). These lines of evidence indicate that under HI conditions, neural progenitor cells (NPCs) in the SVZ proliferate and generate new neuronal cells in response to injury and may enhance neural repair.

Microglia are resident innate immune cells and are the first defense mechanism and mediators of inflammation in the central nervous system (Clarkson et al., 2005[6]; Perlman, 2007[32]; Lai and Yang, 2011[21]; Cerio et al., 2013[4]; Kohman and Rhodes, 2013[20]; Wolf et al., 2017[53]). Microglia also play essential roles in brain development (Tremblay et al., 2011[46]; Tay et al., 2019[44]). In postnatal brain development, microglia regulate synapse development and maintenance (Paolicelli et al., 2011[31]; Schafer et al., 2012[36]), axonal path finding (Squarzoni et al., 2014[42]), and cortical layer formation (Ueno et al., 2013[47]; Squarzoni et al., 2014[42]). Microglia are also present in the prenatal brain, where they regulate cortical cell production in the developing brain through phagocytosis of neural precursor cells (Cunningham et al., 2013[7]). In addition, *in vivo *studies provide evidence that microglia play a supportive role by triggering the expression of neurotrophic factors that promote the survival of NPCs (Johnston et al., 2001[19]; Clarkson et al., 2005[6]; Whitney et al., 2009[52]; Cerio et al., 2013[4]; Kohman and Rhodes, 2013[20]). Specifically, previous work has indicated that microglia can exert proneurogenic effects following hypoxic injury (Thored et al., 2009[45]). The present study investigated the direct impact of microglial function on NPCs proliferation under HI conditions through *in vitro* coculture of NPCs with microglia. Understanding the influence of microglial cells on NPCs proliferation and survival following HI insult may pave the way for future research on neural repair by targeting the function of microglial cells.

## Materials and Methods

### Animals and experimental design

**Figure 1:**
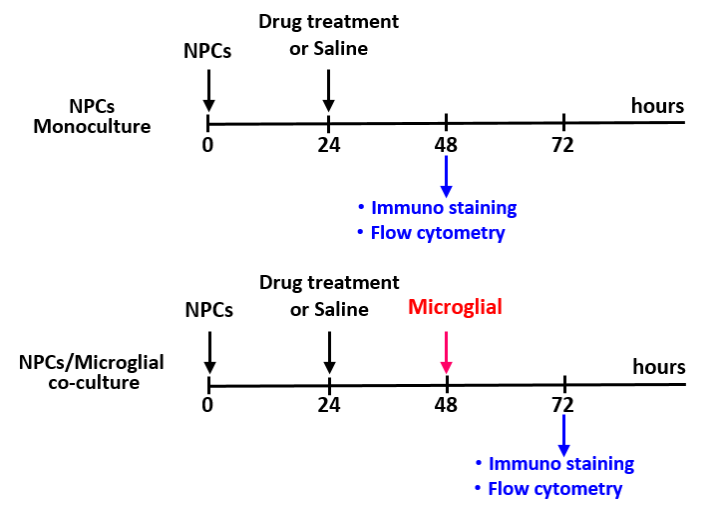
Schematic diagram illustrating the experimental design.

Pregnant C57BL/6 mice weighing between 20-30 g were used to prepare primary NPCs cultures. Animals had free access to food and water and were maintained on a 12-hour light/dark cycle. On gestational day 14, the embryos were dissected, and NPCs were harvested from the cerebral cortex for primary cultures. Brain tissues from newborn mice were harvested to prepare primary microglial cultures. The primary cultures were divided into four groups as follows: 1) normoxic NPCs (NN); 2) normoxic NPCs coculture with microglia (NN+M); 3) hypoxic NPCs (HN); and 4) hypoxic NPCs coculture with microglia (HN+M). Tissues from all culture conditions were used for immunofluorescence staining, western blot analysis, and flow cytometric analysis of the cell cycle. The experimental design is shown in Figure 1(Fig. 1).

Every effort was taken to minimize the number and suffering of animals used for these experiments. All animal procedures were approved by the Institutional Animal Care and Use Committee (IACUC) of the University of California, Davis, and the IACUC of the Institute of Molecular Biosciences, Mahidol University (COA NO. IMB-ACUC 2020/008).

### Primary NPCs/microglial cocultures

Primary NPCs cultures were obtained from the cerebral cortices of C57BL/6 mice on embryonic day 14 (E14). The brain tissues were carefully stripped of the meninges and digested with Accutase for 5 minutes at 37 °C. Cells were centrifuged for 5 minutes at 1100 rpm and resuspended in culture medium consisting of Neurocult^TM^ basal medium, Neurocult^TM^ proliferation supplement, penicillin/streptomycin, laminin (L2020, Sigma), 10 µg/ml epidermal growth factor (EGF), 10 µg/ml fibroblast growth factor (FGF), and 0.2 % heparin. Then, the cells were seeded in a T75 flask and cultured at 37 °C in a humidified atmosphere with 5 % CO_2_.

Primary microglial cultures were obtained from the primary mixed glial cell culture. The cerebral cortices were dissected from C57BL/6 mouse pups at postnatal day 0 (P0) to P2, carefully stripped of the meninges, and digested with papain (LS003124; Worthington) for 30 minutes at 37 °C. Cells were pelleted (5 minutes, 1100 rpm), resuspended in culture medium consisting of Dulbecco's modified Eagle medium (DMEM) with 10 % fetal bovine serum (FBS) and 1 % penicillin/streptomycin, prepared as a single cell suspension by repeated pipetting and allowing the nondissociated chunks of tissue to settle. The cells in the supernatant were plated into a T75 flask and cultured at 37 °C in humidified atmosphere with 5 % CO_2_ for 7-10 days until shiny and round cells were apparent on the top layer or were floating in the supernatant. Flasks were tapped to detach the cells, and the cells were collected and seeded onto precoated coverslips in 24-well plates to assess microglial purity. The purity of isolated microglial cells exceeded 95 %, as verified by immunocytochemical analysis of the specific microglial marker Iba-1 (anti-ionized calcium-binding adaptor molecule-1). Finally, the culture medium was replaced, and microglial cells were used for coculture experiments with previously prepared NPCs cultures. For cocultures, microglia isolated from primary mixed glial cells were collected and seeded on top of NPCs at a density of 2.5× 10^4^ cells/well for 24 hours before analysis.

### Mimetic hypoxia

Mimetic hypoxia was induced by the hypoxia mimetic agent DFO (D9533; Sigma). To determine the optimal concentration and the appropriate incubation time for DFO-induced hypoxia, NPCs were seeded on poly-l-ornithine-coated coverslips in 24-well plates at a density of 1× 10^5^ cells/well for 24 hours, and NPCs were treated with DFO at a final concentration of 0, 25, 50, or 100 μM for 3, 6, 12, and 24 hours. The optimal concentration was chosen by analyzing cell viability.

### Cell viability assay

The vital dye MTT (3-(4,5-dimethyl-thiazol-2-yl)-2,5-diphenyl-tetrazolium bromide) was used to assess cell viability. Living cells take up MTT dye, which is converted from yellow to dark blue formazan crystals by cellular dehydrogenase. MTT was prepared as a 5 mg/ml stock solution in phosphate-buffered saline (PBS) and kept in the dark. The MTT solution (working solution) was added to cultured cells and incubated at 37 °C for 4 hours, and then dimethyl sulfoxide was added to dissolve the formazan crystals. The optical density of the formazan solution was read by an automated microplate reader (SpectraMax) at a spectral wavelength of 570 nm.

### Western blot analysis

The expression of HIF-1α was examined by western blot analysis. Cellular proteins were extracted and homogenized in lysis buffer containing a phosphatase inhibitor. The supernatant was isolated from the lysate, and the protein concentration was measured using a Bradford assay. Thirty micrograms of protein was resolved by electrophoresis on 10 % acrylamide gels and electrotransferred onto a PVDF membrane. The membrane was incubated in blocking buffer for 1 hour, incubated with mouse monoclonal anti-HIF-1α (sc-53546, 1:100) overnight, rinsed 3 times in Tris-buffered saline and 0.1 % Tween 20 (TBST), and then incubated with donkey anti-mouse secondary antibody for 1 hour. A chemiluminescent reagent (ECL; Amersham Biosciences, Piscataway, NJ, USA) was used to detect the signal, and images were scanned by the Azure Biosystems imaging system. ImageJ (National Institutes of Health, USA) was used for quantitative analysis by normalization to β-actin as a loading control.

### Immunofluorescence staining

Cultured cells in each group were fixed with 4 % paraformaldehyde in PBS for 15 minutes at room temperature. Cells were permeabilized with 0.2 % Triton X-100 in PBS for 10 minutes, and nonspecific staining was eliminated by incubation with a blocking buffer containing 10 % donkey serum with 0.2 % Triton X-100 for 2 hours at room temperature. The cells were incubated with the following primary antibodies; rabbit polyclonal anti-Iba1 (019-19741, 1:500), mouse monoclonal anti-Sox2 (MAB2018, 1:50), and anti-phospho Histone H3 (Ser10) PH3 (06-570; 1:400) at 4 °C overnight, rinsed 3 times in PBS and incubated with appropriate secondary antibodies for 2 hours at room temperature. Primary and secondary antibodies were diluted in blocking buffer. Finally, the cells were mounted with Mowiol on a glass slide and observed under a confocal microscope (Olympus Fluoview). All images were taken in a randomized order, and the data were analyzed by a blinded observer. The images were taken at 10x magnification in a minimum of three random fields per coverslip in triplicate. The number of immunopositive cells stained with each antibody was counted by ImageJ in an area measuring 0.015 mm^2^ per image.

### Flow cytometric analysis of the cell cycle

Cell cycle analysis was performed by BD FACSCanto^TM^ flow cytometer (Becton Dickinson (BD), San Jose, CA, USA) using propidium iodide/RNase (PI/RNase) staining buffer (BD Biosciences Pharmingen, USA). PI/RNase was added to the cell culture after fixation with 70 % ethanol overnight. The PI signal was detected using a 562-588 nm bandpass filter.

### Statistical analysis

Statistical analysis was performed using GraphPad Prism software 7.0, USA. The results are expressed as the mean ± SEM. Significance was assessed via a one-tailed, paired Student's t-test. Data quantification among the four groups was compared using one-way analysis of variance (ANOVA) followed by Tukey's multiple comparisons test. Probability values of *p<*.05 were considered statistically significant.

## Results

### The hypoxia mimetic agent DFO induced HIF-1α expression in primary NPCs cultures

**Figure 2:**
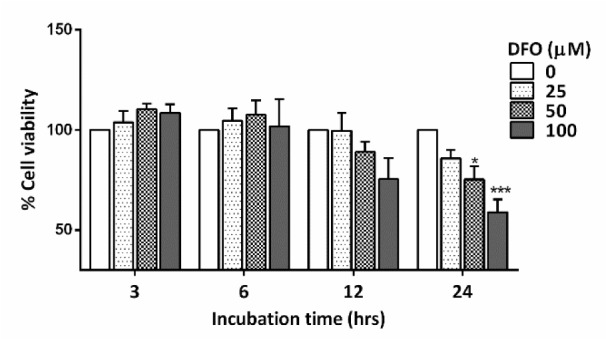
Bar graph comparing the % cell viability of primary NPCs treated with various concentrations of DFO for 3 to 24 hours (n=3 for each group). The data were obtained from three independent experiments. The values represent the mean ± S.E.M. ^*^p<.05 and ^***^p<.001 compared to the untreated group

**Figure 3:**
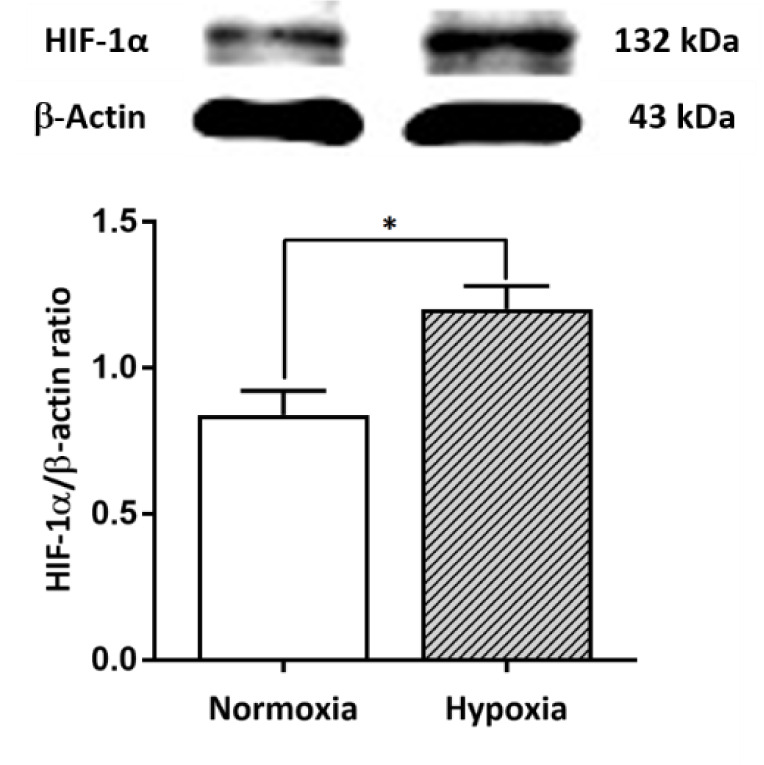
Effect of the hypoxia mimetic agent DFO on HIF-1α protein expression in primary NPCs cultures. Upper panel: Western blot analysis of HIF-1α protein in the normoxic and hypoxic groups. Lower panel: The bar graph displays the quantitative results from three independent experiments. The data are expressed as band densities/β-actin ratios. The values represent the mean ± SEM, n=3 per group. ^*^p<.05 compared with the normoxic group

NPCs harvested from E14 C57BL/6 mice were seeded onto 96-well plates as primary cultures. Hypoxia-ischemia was induced in NPCs by DFO at various concentrations and for different incubation times to determine the optimal conditions for further experiments. A final concentration of 100 µM DFO and 24 hours of incubation were selected based on significantly reduced cell viability (*p<*.001) (Figure 2(Fig. 2)). The working dose of DFO used in the present study corresponded to that used in previous studies, which reported that 100 µM DFO could induce cell death in primary neuronal cell cultures (Walls et al., 2009[50]; Salmaso et al., 2014[35]). In the present study, NPCs treated with 100 µM DFO for 24 hours showed a significant increase in HIF-1α expression (*p<.*05) compared to that of controls (Figure 3(Fig. 3)). The results indicate that DFO is an effective hypoxia mimetic agent for inducing HI injury, as indicated by a significant increase in the level of HIF-1α.

### Microglia enhanced NPCs maintenance and survival under hypoxic-ischemic conditions

**Figure 4:**
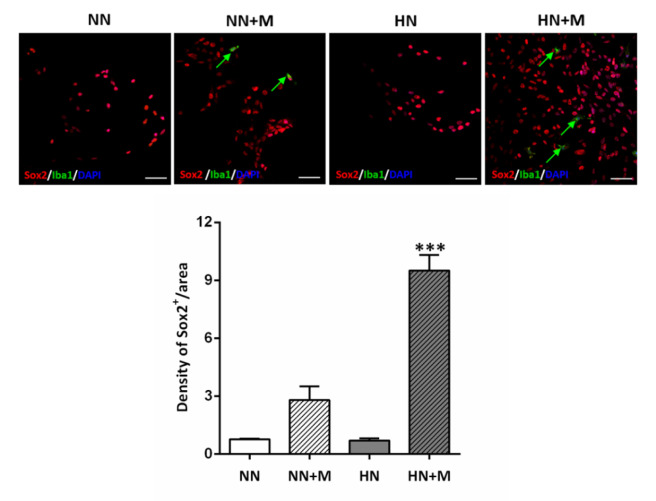
Effect of microglia on the density of Sox-2-positive NPCs under normoxic and hypoxic conditions. Upper panel: Photomicrographs of immunofluorescence staining of Sox2 (red), Iba1 (green), and DAPI (blue). Arrows indicate Iba1-positive microglial cells. Scale bar = 20 µm. Lower panel: Bar graph comparing the density of Sox2-positive cells in the four groups. The data were obtained from three independent experiments. The values represent the mean ± SEM, n=3 per group. ^***^p< .001 compared with the other groups. (Notes: NN = normoxic NPCs, NN+M = normoxic NPCs + microglia, HN = hypoxic NPCs, HN+M = hypoxic NPCs + microglia)

To evaluate NPCs survival and maintenance following hypoxic injury, NPCs cultures from all groups were stained with the anti-Sox2 and anti-Iba1 primary antibodies, and DAPI. The results showed that there was no significant difference in the number of Sox2-positive cells between normoxic and hypoxic conditions. However, we noted a significant increase in the number of Sox2-positive cells in the HN+M coculture group compared to the HN group (Figure 4(Fig. 4)). We noted an increasing trend in the number of Sox2-positive cells in the NN+M group, but it was not statistically significant.

### Microglia promoted cell proliferation under hypoxic-ischemic conditions

**Figure 5:**
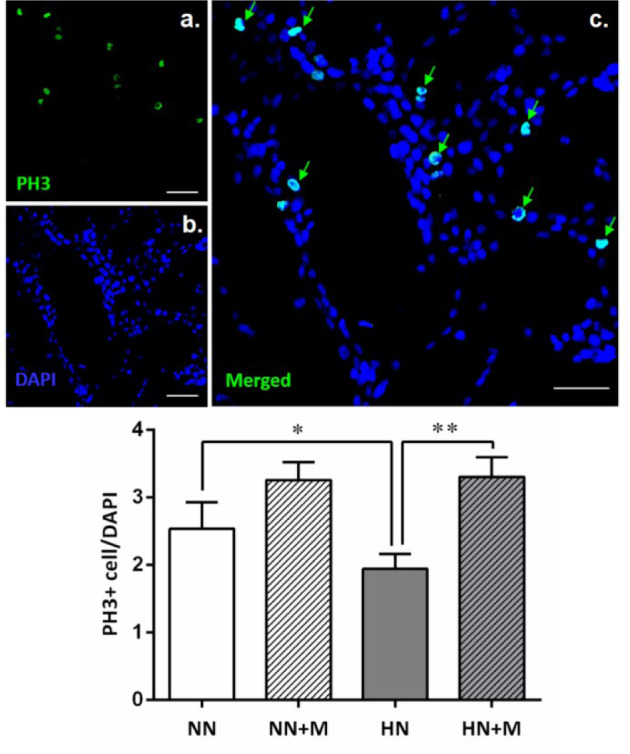
Effect of microglia on the number of PH-3-positive NPCs under normoxic and hypoxic conditions. Upper panel: Photomicrographs of immunostaining of NPCs for the mitosis marker PH3 (green) and DAPI (blue). Green arrows indicate double staining of PH3 and DAPI, which represent dividing NPCs. Scale bar = 20 µm. Lower panel: Bar graph comparing the number of PH3/DAPI double-stained NPCs in the four groups. The data were obtained from three independent experiments. The values represent the mean ± SEM, n=3 per group. ^*^p<.05 compared with the normoxic groups, ^**^p<.01 compared with the hypoxic groups. (Notes: NN = normoxic NPCs, NN+M = normoxic NPCs + microglia, HN = hypoxic NPCs, HN+M = hypoxic NPCs + microglia)

The proliferation of NPCs was examined by immunostaining with the mitotic marker PH3. The results showed a significant decrease in proliferation after HI treatment; the number of PH-3/DAPI double-positive cells in the HN group was significantly decreased compared to that in the NN group (^*^p<.05) (Figure 5(Fig. 5)). Interestingly, the presence of primary microglia reversed this effect by increasing the number of PH-3/DAPI double-positive cells in the HN+M group compared to the HN group (^**^p<.01). Among the normoxic groups, although there was a small increase in the number of PH-3/DAPI double-positive cells in the NN+M group, this increase was not significantly different compared to that of the NN group. These results indicate that microglia can enhance NPCs proliferation following HI injury.

**Figure 6:**
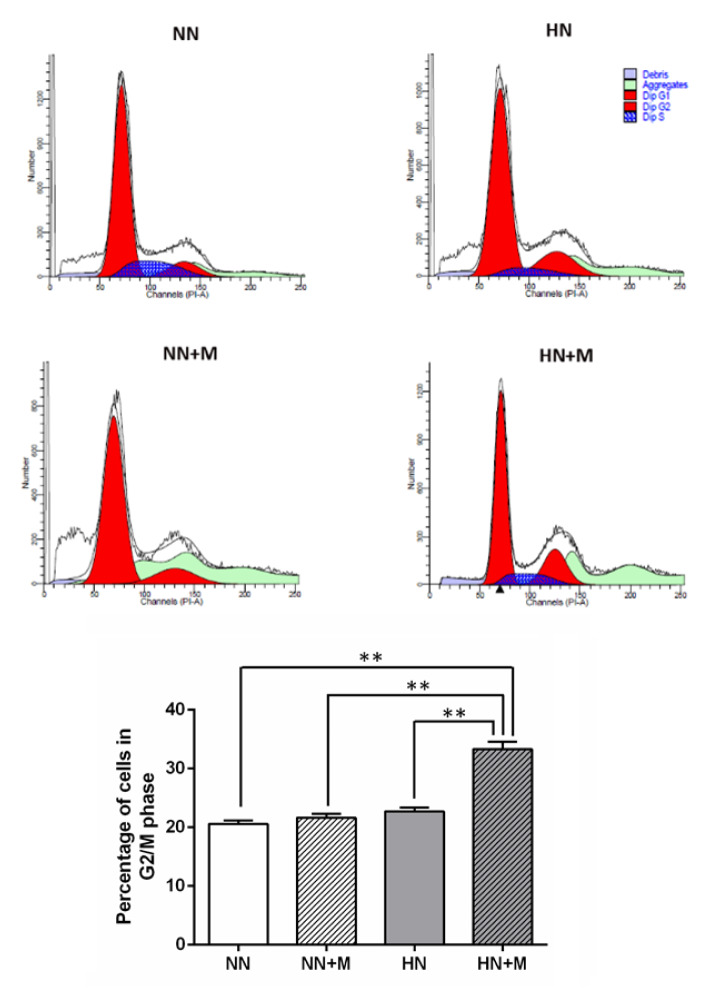
Effect of microglia on NPCs proliferation under normoxic and hypoxic conditions. Upper panel: Flow cytometric analysis of the cell cycle phases compared among the four groups. The cell cycle of proliferating NPCs was detected by PI/RNase staining, and the percentages of cells in the G1, S, and G2/M phases were analyzed with ModFit software. Lower panel: Bar graph comparing the number of NPCs in the G2/M phase of the cell cycle across the four groups. The data were obtained from three independent experiments. The values represent the mean ± SEM, n=3 per group. ^**^p< .01 compared with the other groups. (Notes: NN = normoxic NPCs, NN+M = normoxic NPCs + microglia, HN = hypoxic NPCs, HN+M = hypoxic NPCs + microglia)

We next used flow cytometry to determine the percentage of cells in each phase of the cell cycle after HI. The dynamics of the cell cycle were detected by PI/RNase staining, and the percentage of cells in each phase (G1, S, and G2/M) was analyzed with ModFit software. The results showed that there was no significant difference in the percentage of cells in G1 and S phase among all groups. However, there was a significant increase in the percentage of cells in the G2/M phase in the HN+M group compared with the other groups (Figure 6(Fig. 6)).

## Discussion

Hypoxic ischemia is estimated to occur in 1 to 9 per 1,000 live births and in an ever greater proportion of premature births (Gopagondanahalli et al., 2016[14]). Some studies have reported increased incidences in different regions of the world, which may impact more than three percent of the population (Namusoke et al., 2018[27]). Improved treatment for this condition relies on a better understanding of how distinct populations of brain cells react to hypoxia. We examined this issue using primary cell cultures prepared from embryonic and neonatal brain cells using a model of HI injury. The hypoxia mimetic agent DFO is commonly used to induce hypoxia *in vitro* (Wu and Yotnda, 2011[54]). We found that 100 µM DFO induced cell death in nearly 40 % of primary NPCs and significantly increased the expression of the hypoxia indicator HIF-1α. Our findings corresponded to previous reports that DFO induced HIF-1α expression at both the gene and protein levels: cells in the hypoxic group showed elevated levels of HRE (hypoxia-responsive element) and HIF-1α compared to those of the normoxic group (Guo et al., 2006[16]; Elstner et al., 2007[10]; Milosevic et al., 2009[25]; Li et al., 2014[22]). Taken together, our results and previous studies demonstrate that DFO is an effective hypoxia mimetic agent for inducing HI injury *in vitro*.

HIF-1α plays dual roles as a prodeath or prosurvival signal, depending on the duration and severity of hypoxia (Shingo et al., 2001[40]; Fan et al., 2009[11]; Shi, 2009[39]; Giese et al., 2010[13]; Harms et al., 2010[17]). For example, under mild hypoxic conditions, HIF-1α induces the expression of prosurvival genes, such as erythropoietin (EPO) and vascular endothelial growth factor (VEGF), thereby enhancing neovascularization and neuronal survival. In contrast, under severe HI injury, HIF-1α activates prodeath genes, such as BNIP (BCL2/adenovirus E1B 19 kD-interacting protein) and p53, leading to neuronal cell death.

During pre- and postnatal brain development, microglia interact reciprocally with NPCs (Barger et al., 2019[2], Noctor et al., 2019[29]). On the one hand, microglia regulate various processes associated with brain development, such as NPCs proliferation, survival, and differentiation. On the other hand, NPCs regulate microglial function and activity by secreting proteins that modulate microglial activation and proliferation (Mosher et al., 2012[26]). This reciprocal relationship may persist throughout life (Su et al., 2014[43]). Following stroke, microglial cells are a part of the endogenous brain defense mechanism that helps reduce neuronal damage and promote tissue regeneration and repair (Faustino et al., 2011[12]).

Previous studies have provided evidence that NPCs functions are enhanced following certain types of brain insult (Abraham et al., 2001[1]). For example, *in vivo* studies have shown that new neuronal cells are produced in the SVZ following brain injury (Ong et al., 2005[30]). Many of the newly generated neurons migrated to the site of injury within the striatum, and following stroke, microglia within the SVZ increased the expression of insulin-like growth factor 1 (IGF-1) (Thored et al., 2009[45]). *Ex vivo* studies also provide evidence that microglia are activated and secrete factors to promote neural stem/ progenitor cell proliferation, survival, and differentiation following brain injury (Deierborg et al., 2010[9]). These lines of evidence from both *in vivo* and *ex vivo* studies show that microglia play a pivotal role in protecting against neuronal injury by enhancing the proliferation, survival, and differentiation of NPCs. However, direct evidence that microglia enhance NPCs proliferation and survival following HI injury is scarce.

We used an *in vitro* coculture model of NPCs and microglial cells to further test the hypothesis that microglia enhance NPCs proliferation and differentiation following hypoxic injury. We found that microglia enhance NPCs proliferation following HI injury, as evidenced by a significant increase in the percentage of cells in the G2/M phase, with no change in the percentage of cells in the S or G1 phase of the cell cycle. We also found that the number of PH3- and Sox2-positive cells was significantly increased in hypoxic NPCs cocultured with microglia. Taken together, these results indicate that microglia increase NPCs proliferation, survival, and differentiation under hypoxic conditions.

Our results are consistent with previous studies showing that new neurons are produced in the SVZ of the immature brain following HI injury and that the newly generated neurons enter the neocortex together with large numbers of activated microglia (Yang et al., 2007[56]). The colonization of the cortex was associated with increased expression of growth factors (i.e., IGF-1) and chemokines (i.e., monocyte chemoattractant factor-1 or CXCL12) in neocortical neurons that was sustained for months (Yang et al., 2007[56]). Another study reported that stroke alters the dynamics of the cell cycle kinetics of NPCs in the SVZ. For example, NPCs proliferation in the SVZ was increased within two days following stroke and reached a maximum level at 4 to 7 days following perinatal HI injury (Zhang et al., 2008[59]). Changes in cell cycle kinetics included shortening of the G1 phase without affecting the G2, M, and S phases of the cell cycle (Zhang et al., 2008[59]). These data suggest that HI increase NPCs proliferation by shortening the length of the cell cycle, leading to increased production of new neurons.

Microglial activation in the CNS is often classified along a spectrum, with the M1 phenotype, which is considered neurotoxic, at one end of the axis, and the M2 phenotype, which is considered neuroprotective, at the opposite end of the axis. In animal models of ischemic brain injury, the M2 phenotype produces anti-inflammatory cytokines that stimulate the proliferation and differentiation of endogenous neural stem cells (Choi et al., 2017[5]). When brain sections prepared from animals that experienced transient middle cerebral artery occlusion were cultured with M2-conditioned medium, the mRNA levels of transforming growth factor-alpha (TGF-α) and IL-10 were significantly increased, and markers of cell division were also increased (Yuan et al., 2017[58]). These lines of evidence from *in vivo* and *ex vivo* models point to a role for microglia in stimulating NPCs proliferation following HI. Our results provide further evidence for a protective and promoting role of microglia on NPCs proliferation and differentiation in an *in vitro* model of NPCs/microglia cocultures.

The signaling mechanisms by which microglia promote NPCs proliferation have been identified. First, M2 microglia secrete the chemokine PPARγ (peroxisome proliferator-activated receptor γ), which upregulates epidermal growth factor receptor (EGFR) and cyclin B, to promote NPCs proliferation (Wada et al., 2006[49]; Yuan et al., 2017[58]). In addition, activation of PPARγ inhibits the expression of proinflammatory cytokines, such as nitric oxide and TNF-α, and promotes the differentiation of immune cells towards an anti-inflammatory phenotype (Martin, 2010[23]; Song et al., 2016[41]). Second, evidence indicates that CXCL12 signaling occurs between NPCs and microglia. During NPCs proliferation, cortical progenitor cells produce stromal cell-derived factor-1 (also called CXCL12), while microglia express the receptors Cxcr4 and Cxcr7 to regulate the cell cycle. CXCL12 signaling increases the protein expression of cyclin D1, which is required for progression through the G1 phase of the cell cycle. In this way, activation of the CXCL12 pathway leads to shortening of the G0/G1 phase while lengthening the S phase of the cell cycle (Wang et al., 2016[51]). Third, recent *in vitro* studies of NPCs/microglia cocultures reported that microglia-induced proliferation might act through osteopontin (OPN) receptor signaling, since inhibition of the OPN receptor reduced microglial-induced proliferation (Yamamiya et al., 2019[55]). Further study will be required to explore the mechanisms by which microglia influence NPCs proliferation under HI conditions.

## Conclusion

In conclusion, our results show that following HI injury, microglia increase NPCs proliferation, survival, and differentiation, thereby potentially enhancing the ability of newly generated neurons to repair affected tissues. The neuroprotective effects of microglia may be a strategic target for novel therapies to treat HI injury in the immature brain.

## Acknowledgements

This work was supported by Thailand Research Fund (TRF) under the TRF-RSA Research Grant (RSA57) to Nuanchan Chutabhakdikul; the TRF-RGJ Ph.D. Program in collaboration with the Huachiew Chalermprakiet University (PHD/0056/2555) to Supanee Chounchay, and the NIH grant (MH101188) to Stephen C. Noctor.
